# How to innovate and make successful business

**DOI:** 10.1186/2193-1801-4-S2-K2

**Published:** 2015-11-27

**Authors:** Eric Chi-ming Yim

**Affiliations:** POSH Office Systems (HK) Limited, Hong Kong; The Council, Vocational Training Council, Hong Kong

Nowadays, facing the rapidly changing economic and business environment, growing competition from competitors and rising consumer expectations, enterprise which has withstood various tests of changeable situations, "innovation" is the way out amid fierce competition and one of the keys to success. Innovation means implementing new ideas, developing new products and improving existing services. Innovation helps enterprise grow in the marketplace and make business success.

Innovation is ubiquitous. Based on my experiences in the office furniture industry, there are three major areas of innovation: product innovation, organisational innovation and marketing innovation. They are closely related to each other.

## Product innovation

Many successful enterprises are strong at product innovation which helps to meet customer expectations and keep abreast or ahead of competitions. Product innovation always boosts the upgrading and transformation of an enterprise. To strengthen product differentiation, we must continually develop new product, improve product design, and use new technology and environmental friendly materials. Due to the severe competition in office furniture industry, POSH focuses more on "value" rather than "price". In the past, traditional steel office furniture was static and bulky. Today, POSH pays more attention to the aesthetic value, creates functions of furniture in various styles. Besides, our furniture are smartly designed taking into account the environmental issues, such as encourage waste reduction, the use of recycled material and improve disposal methods of waste generated in production. As such, our products are not only ergonomically designed for comfort and health but also solving the environmental, education, housing, logistics and transportation and other social problems. Our staff members were trained and empowered to serve customers by "Better Design, Better Environment". Given the rapid ageing of the Hong Kong population, I believe that more enterprises will proceed with designing products catering for the elderly and were innovative and of high quality to grasp the opportunity.

## Organisational innovation

Organisational innovation is prerequisite of successful business. In order to excel in the highly competitive market, enterprises must ensure their business and management models and vocational education and training programmes move with the times. Business leaders should equip themselves with innovative leadership, and capitalise on their own strengths while harnessing the capabilities and assets of others with a view to climbing to the top of the market. Being the CEO and the Chief Designer of POSH, I ran the business and made recommendations from multiple perspectives. As early as the 1990s, POSH had entered a dealership arrangement with Herman Miller to market its products in Hong Kong and mainland China. In 2008, POSH formed an international strategic alliance with Herman Miller to bring about mutual benefits. POSH assisted Herman Miller in tapping the booming mainland market, meanwhile, POSH used Herman Miller as the gateway to grow its international business. Besides, in 1997, POSH had developed a new franchise concept in office furniture industry in Mainland and explored to expand into international markets. Today, POSH has over 20 retail outlets in major cities of the mainland, including Shanghai, Beijing, Guangzhou and Shenzhen.

## Marketing innovation

Marketing innovation is vital to achieve success in today's evolving marketplace. Brand building and market positioning are crucial factors for the success. In the ever-changing global market, enterprises have to develop innovative design and brand name to add value and enhance market competitiveness. At the inception of POSH, I had started my original design manufacturing (ODM) and original brand manufacturing (OBM) business. I registered POSH's trademarks both in the mainland and Hong Kong to make the brand unique, enhance product value and boost consumers' confidence. Besides, POSH adopted a case study approach to brig innovative solutions and new technologies to help customers optimising work efficiency and increasing the productivity while maintaining mobile connectivity and environmental sustainability. This approach assisted our enterprise in gaining a competitive advantage and expanding the market.

